# A CTR prediction model based on session interest

**DOI:** 10.1371/journal.pone.0273048

**Published:** 2022-08-17

**Authors:** Qianqian Wang, Fang’ai Liu, Xiaohui Zhao, Qiaoqiao Tan

**Affiliations:** 1 Shandong Women’s University, Jinan, China; 2 Shandong Normal University, Jinan, China; Taipei Medical University, TAIWAN

## Abstract

Click-through rate prediction has become a hot research direction in the field of advertising. It is important to build an effective CTR prediction model. However, most existing models ignore the factor that the sequence is composed of sessions, and the user behaviors are highly correlated in each session and are not relevant across sessions. In this paper, we focus on user multiple session interest and propose a hierarchical model based on session interest (SIHM) for CTR prediction. First, we divide the user sequential behavior into session layer. Then, we employ a self-attention network obtain an accurate expression of interest for each session. Since different session interest may be related to each other or follow a sequential pattern, next, we utilize bidirectional long short-term memory network (BLSTM) to capture the interaction of different session interests. Finally, the attention mechanism based LSTM (A-LSTM) is used to aggregate their target ad to find the influences of different session interests. Experimental results show that the model performs better than other models.

## 1. Introduction

The prediction of click-through rate (CTR) is a critical problem on ads or items for many applications such as online advertising or recommender systems [1,2]. It is to estimate the probability a user will click on a recommended item. Cost per click (CPC) [3] model is often used in advertising system. The accuracy of click-through rate (CTR) can influence the final revenue in CPC model. In many recommendation systems, the goal is to maximize the number of clicks, so recommended items can be ranked by estimated CTR.

It is important for CTR prediction to find feature interactions based on user behavior. However, most models ignore to capture user interest behind user behavior. User interest has an important influence on CTR prediction. In the fields with rich internet-scale user behavior data, such as online advertising, user sequential behaviors reflect user evolving interests. Some researchers overlook the intrinsic structure of the user behavior sequences. Multiple sessions make up a sequence. A session is a list of user behaviors that occur within a given time frame. The user behavior in each session is highly homogeneous, and the user behavior in different sessions is heterogeneous. Grbovic et al. [4] found the session division principle that there is a time interval of more than 30 minutes. As we can know, the user mainly browses the shoes in the first half an hour as session 1, and browses the watch in the second half an hour as session 2. It is the fact that people has a clear and unique intent at a session, but the interest usually changes when user start a new session.

Through the above observation, we propose a hierarchical model based on session interest (SIHM) for CTR prediction, which uses multiple historical sessions to simulate the user’s sequential behavior in the CTR prediction task. At session division module, we naturally divide the user sequential behavior into sessions. At session interest extractor module, we apply a self-attention mechanism with bias coding to model each session. Self-attention mechanism gets the internal relationship of each session behavior. Since different session interest may be related to each other or follow a sequential pattern, so we chooses a bidirectional long short-term memory (BLSTM) network [5] to model the dependency between session interest at session interacting module. The auxiliary tasks are employed for producing the interest state with the deep supervision strategy to learn the current hidden state. It can help the model learn more interest-encoded latent representation and enforce the hidden state to capture the session interest. Because different session interests have different effects on the target item, we utilize attention mechanism to achieve local activation and use LSTM to aggregate their target ad to get the final representation of the behavior sequence.

The main contributions of this paper are as follows:

1. The user behavior in each session is highly homogeneous, and the behavior of user in different sessions is heterogeneous. We focus on user multiple session interest and propose a hierarchical model based on session interest (SIHM) for CTR prediction. We can get more expressions of interest and more accurate prediction results.
2. To effectively capture session interest, we devise session interest extractor module and divide the user sequential behavior into sessions. We employ a self-attention network obtain an accurate expression of interest for each session. The auxiliary tasks are employed for producing the interest state with the deep supervision strategy to learn the current hidden state. We use BLSTM to capture the interaction of different session interests. Then the attention mechanism based LSTM (A-LSTM) is used to aggregate their target ad to find the influences of different session interests at session interacting module.
3. The experimental results demonstrate that our proposed model has great improvements over other models. In addition, we explore the impact of key parameters, which proves the validity of the SIHM model.

This work is organized as follows. In Section 2 we discuss the related work and introduce the detailed architecture of proposed SIHM model in Section 3. Then we verify the prediction effectiveness of the proposed model in Section 4. Furthermore, in Section 5, we summarize the model presented in this paper and introduce the direction of future work.

## 2. Related work

There are many models proposed by researchers for CTR prediction as a binary classification problem. Logistic regression (LR) [6] is a linear model that is used in the industry. Some researchers established models based on LR [7] for CTR prediction. Jiang et al. [8] introduced a model named SAE-LR to extract the abstract features and got better performance than LR. The advantages of linear models are simplicity and portability, but are weaker in capturing feature interactions. To overcome the limitation, Factorization Machine [9] (FM) and its variants [10] are used to capture feature interactions. The field-aware factorization machines (FFM) introduced field aware latent vectors to capture feature interaction. Liu et al. [11] proposed a FPENN model that combined field-aware embedding and high-order feature interactions. However, most models use shallow layer that have limited representation power of feature interactions.

Recently, due to the powerful ability of feature representation, deep neural networks have achieved great success in many research fields such as in computer vision [12,13], image identification [14,15] and natural language processing [16,17]. Therefore, different kinds of deep neural networks are applied to CTR prediction. Chen et al. [18] combines together the powerful data representation and feature extraction capability of Deep Belief Nets, with the advantage of simplicity of traditional Logistic Regression models. Zhang et al. [19] proposed the Factorization Machine based Neural Network (FNN). The model uses FM to pre-train the embedding layer based on forward neural network. DeepFM model [20] uses FM to replace the wide part, and shared the same input. DeepFM model is considered to be the more advanced model in the field of CTR estimation. Product-based Neural Networks (PNN) model [21] is used for user response prediction. The model utilizes a product layer and gets feature interaction. Zhou et al. [22] proposed a DGRU model, which integrates DeepFM and GRU to improve the accuracy of prediction. Convolutional Neural Network (FGCNN) [23] model was introduced to solve the problem of feature interaction. The model leverages the strength of CNN to generate local patterns and recombine them to generate new features. Huang et al. [24] introduced a new model based on Deep&Cross Network [25], the model can get better feature interaction. Cross Network [26] further replaces the cross vector in Cross Network into a cross matrix to make it more expressive. Convolutional Neural Networks (CNN) and Graph Convolutional Networks (GCN) are also explored for feature interaction modeling. Convolutional Click Prediction Model (CCPM) [27] performs convolution, pooling and non-linear activation repeatedly to generate arbitrary-order feature interactions. However, CCPM can only learn part of feature interactions between adjacent features since it is sensitive to the field order. Feature Generation Convolutional Neural Network (FGCNN) improves CCPM by introducing a recombination layer to model non-adjacent features [28]. It then combines the new features generated by CNN with raw features for final prediction. Early deep CTR models alleviate human efforts in feature engineering by incorporating simple MLPs.

In practical applications, different predictors usually have different predictive capabilities. Features that have a greater contribution to the prediction results should be given greater weights. As we all know, the attention mechanism [29] has a powerful function in distinguishing importance of features. Zhang et al. [30] proposed a novel framework called Multi-Scale and Multi-Channel neural network (MSMC) to learn the feature importance and feature semantics for enhancing CTR prediction. Wang et al. [31] improves FM based on the attention mechanism to find the different importance of different features. Zhang et al. [32] proposed a deep CTR prediction model based on attention mechanism, which can make use of the user historical behavior. High-order Attentive Factorization Machine (HoAFM) model [33] was proposed based on FM to determine the different importance of co-occurred features on the granularity of dimensions.

In addition to capturing feature interactions, user interest also affects prediction results. Constructing a model to capture the user’s dynamics and evolving interests from the user’s sequential behavior has been widely proven effective in CTR prediction tasks. Deep Interest Network (DIN) model [34] captures user interest from history behavior based DNN. At the same time, Deep Interest Evolution Network (DIEN) [35] was proposed based on DIN. DIEN can not only obtain the user interest features, but also can capture the evolution process of interest. The concept of session often appears in sequential recommendation, but it is rarely seen in CTR prediction tasks. Session-based recommendation achieves good results via user dynamic interest evolving. A personalized interest attention graph neural network (PIA-GNN) was proposed for session-based recommendation used an attention mechanism to capture the user purpose in the current session [36]. Zhang et al. [37] analyzes the current session information from multiple aspects and improves user satisfaction. Session-based recommendation [38] is often used to match the user preferences based on session information. However, most existing studies for CTR prediction ignore that the sequences are composed of sessions. Upon all these perspectives, we introduce a hierarchical model based on session interest (SIHM) to get a better result for CTR.

## 3. Material and methods

We describe SIHM model in this section. We first introduce feature representation and embedding in Section 3.1. Next, Section 3.2 illustrates the session division module. Then, we describe the session interest extractor module in Section 3.3 and session interacting module in Section 3.4. Finally, we present the overall architecture of the SIHM model in Section 3.5.

### 3.1 Feature representation and embedding

We use four groups of features (User Profile, Scene Profile, Target Ad, and User Behavior) as input data for the model. Four groups of features all affect the CTR, but the most important influence on the prediction results is the user behavior feature. We mainly capture the user interest from user behavior feature. The encoding vector of the feature group can be expressed by $E\in {\mathbb{R}}^{M\times d_{\mathrm{model}}}$, where *d*_model_ is the embedding size and *M* is the size of sparse features. Through feature embedding, User Profile can be represented by $X^{U}\in {\mathbb{R}}^{N_{u}\times d_{\mathrm{model}}}$, where *N*_*u*_ is the number of User Profile sparse features. Similarly, both Scene Profile and Target Ad can be expressed as $X^{S}\in {\mathbb{R}}^{N_{s}\times d_{\mathrm{model}}}$, $X^{I}\in {\mathbb{R}}^{N_{i}\times d_{\mathrm{model}}}$, where *N*_*s*_ and *N*_*i*_ are the number of Scene Profile and Target Ad sparse features respectively. User Behavior is represented by $X=[x_{1};\ldots ;x_{i};\ldots x_{N}]\in {\mathbb{R}}^{N\times d_{\mathrm{model}}}$, where *N* is the number of user historical behaviors and *x*_*i*_ is the embedding of the *i*-th behavior.

### 3.2 Session division module

We divide the user behavior sequences X into sessions *S* and get the user session interests, where the *k*-th session $S_{k}=[x_{1};\ldots ;x_{i};\ldots x_{T}]\in {\mathbb{R}}^{T\times d_{\mathrm{model}}}$, *T* is the number of behaviors in each session and b_*i*_ is user *i*-th behaviors in current session. According to Grbovic method, we segment user behaviors more than 30 minutes apart into user sessions.

### 3.3 Session interest extractor module

As far as we know, behaviors in the same session are closely related to each other, and the random behavior of user in a session does not represent the original expression of session interest. We use a multi-head self-attention mechanism [39] to capture the inner relationship between behaviors in the same session and find the impact of those irrelevant behaviors.

Multi-head self-attention can get the relationship in different representation subspaces. We use *S*_*k*_ = [*S*_*k*1_;…;*S*_*kn*_;…*S*_*kN*_], where $S_{kn}\in {\mathbb{R}}^{T\times d_{n}}$ is the *n*-th head of *S*_*k*_. *N* is the number of heads, $d_{n}=\frac{1}{n}d_{\mathrm{model}}$. The output of *head*_*n*_ can be calculated as follows:

$$head_{n}=Attention(S_{kn}W^{Q},S_{kn}W^{K},S_{kn}W^{V})$$ (1)

$$Attention(Q,K,V)=\mathrm{softmax}(\frac{S_{kn}W^{Q}W^{K^{T}}S_{kn}^{T}}{\sqrt{d^{\mathrm{model}}}})S_{kn}W^{V}$$ (2)

where *W*^*Q*^,*W*^*K*^,*W*^*V*^ are weight matrices. Then FNN can further improve the nonlinear ability:

$$I_{k}^{S}=FNN(Concat(head_{1},\ldots head_{N})W^{O})$$ (3)

$$I_{k}=Avg(I_{k}^{S})$$ (4)

where *W*^*O*^ is the weight matrix. *FNN*(⋅) is the feedforward neural network. Avg(⋅) is the average pooling. *I*_*k*_ is the user *k*-th session interest.

### 3.4 Session interest interacting module

We applied the BLSTM module to model the dependency between the different session interest. Each LSTM unit [40,41] maintains a memory *c*_*t*_ at time *t*. One input gate *i*_*t*_ with corresponding weight matrix *W*_*xi*_,*W*_*hi*_,*W*_*ci*_, one forget gate *f*_*t*_ with corresponding weight matrix *W*_*xf*_,*W*_*hf*_,*W*_*cf*_, one forget gate *f*_*t*_ with corresponding weight matrix *W*_*xo*_,*W*_*ho*_,*W*_*co*_.The output *h*_*t*_ of the LSTM unit is then:

$$h_{t}=o_{t}\tanh(c_{t})$$ (5)

where *o*_*t*_ is an output gate that modulates the amount of memory content exposure. The output gate is calculated by the following formula:

$$o_{t}=\sigma (W_{xo}I_{t}+W_{ho}h_{t-1}+W_{co}c_{t}+b_{o})$$ (6)

where *σ* is a logistic sigmoid function.

The *c*_*t*_ denotes the memory cell and it is updated by forgetting irrelevant memory information, and then adding a new memory state ${\tilde{c}}_{t}$:

$$c_{t}=f_{t}c_{t-1}+i_{t}{\tilde{c}}_{t}$$ (7)

where a new memory state can be defined as:

$${\tilde{c}}_{t}=\tanh(W_{xc}I_{t}+W_{hc}h_{t-1}+b_{c})$$ (8)

The forget gate *f*_*t*_ controls the information which the existing memory is forgotten, and the input gate *i*_*t*_ controls the information which the new memory content is added to the memory unit. Gates are computed as follows:

$$f_{t}=\sigma (W_{xf}I_{t}+W_{hf}h_{t-1}+W_{cf}c_{t-1}+b_{f})$$ (9)

$$f_{t}=\sigma (W_{xi}I_{t}+W_{hi}h_{t-1}+W_{ci}c_{t-1}+b_{i})$$ (10)

In bidirectional architecture, there are two layers of hidden nodes from two separate LSTM encoders. The two LSTM encoders capture the dependencies in different directions.

**Fig 1:**
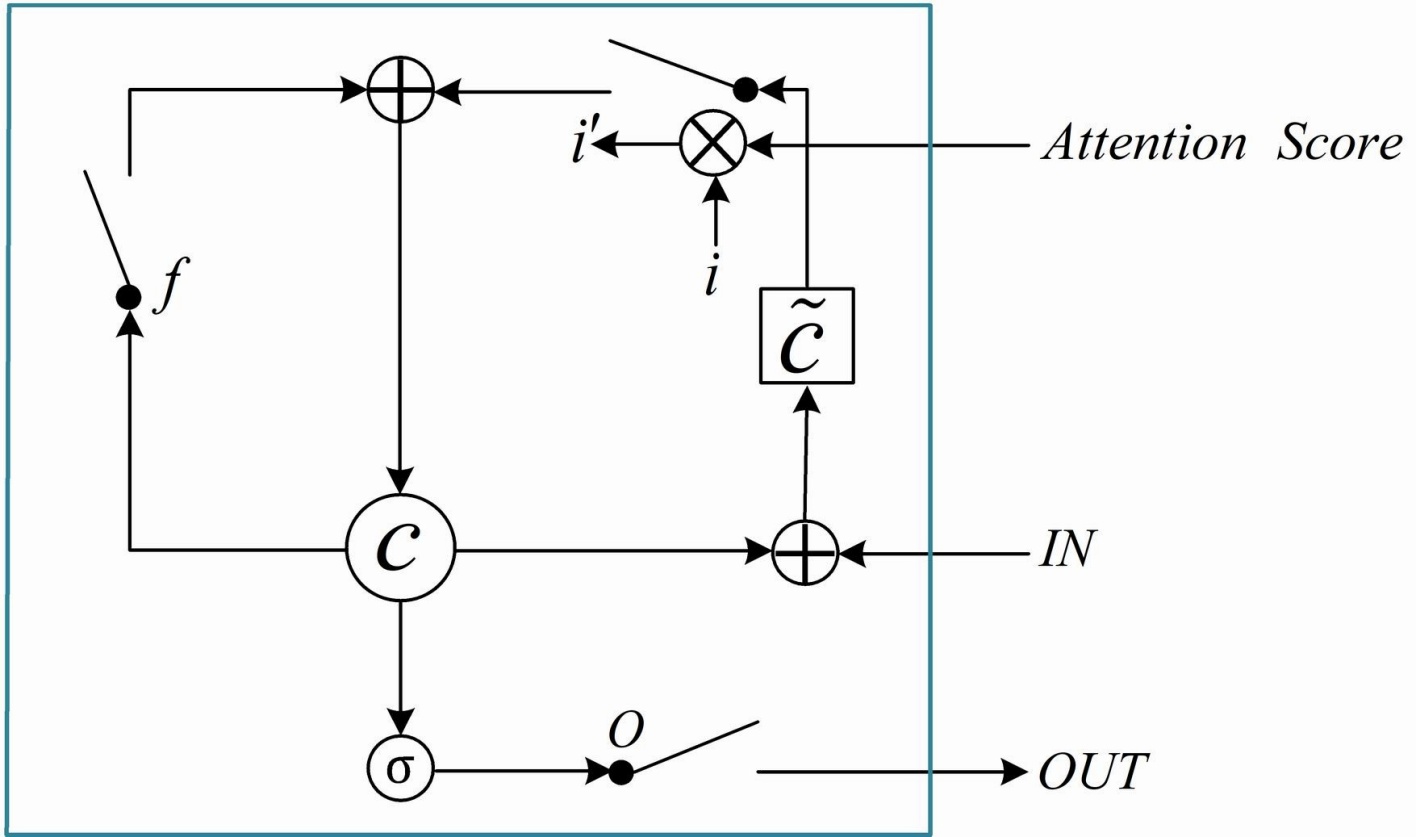
The architecture of A-LSTM.

The hidden state *h*_*t*_ can capture the dependency between session interests. However, the user’s session interest related to the target ad has a greater impact on whether the user will click on the target ad. So the weight of the user’s session interest needs to be reassigned to the target ad. We apply an attention mechanism with LSTM to model the representation of session interests and target ad. Fig 1 shows the framework of the applied attention mechanism with LSTM (A-LSTM).

The $I_{t}^{\prime }$ is the input of the A-LSTM and *h*’_*t*_ is the hidden state. The input to the second A-LSTM can be represented as *I*’_*t*_ = *h*_*t*_. The final interest state is *h’*_*T*_. The attention function is formulated as:

$$a_{k}^{I}=\frac{\exp(I_{k}W^{I}X^{I})}{\sum_{k}^{K}\exp(I_{k}W^{I}X^{I})}$$ (11)

*W*^*I*^ has the corresponding shape, attention score can reflect the relationship between target ad *X*^*I*^ and input.

We use A-LSTM to consider influences of between session interests and the target ad:

$$i_{t}^{\prime }=h_{t}*a_{k}^{I}$$ (12)

where *h*_*t*_ denotes the *t*-th hidden state, *i*′ denotes the entry for the second LSTM module, and the * is the scalar-vector product.

### 3.5 The overall architecture of SIHM model

**Fig 2:**
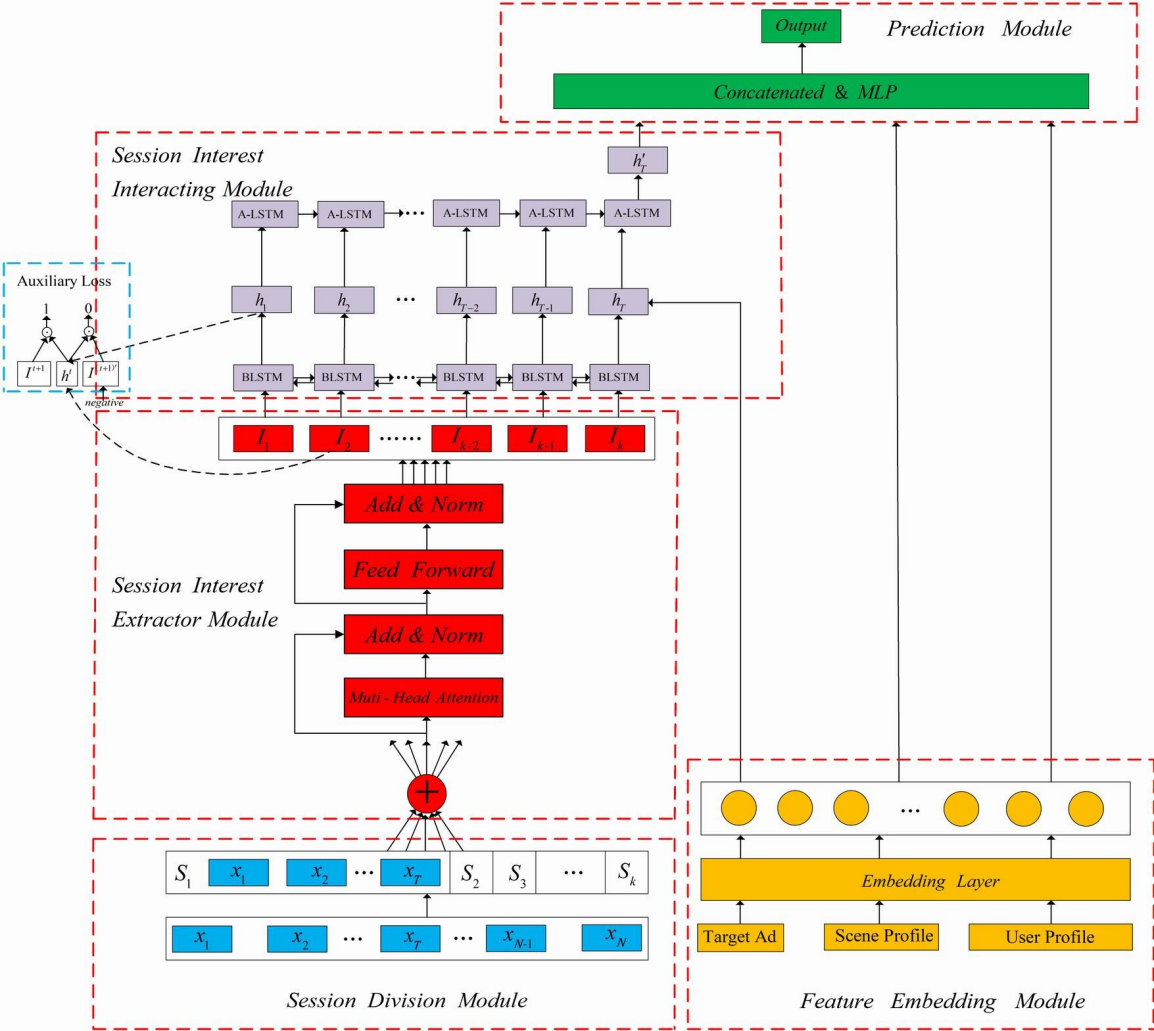
The structure of SIHM.

The structure of the SIHM model is shown in Fig 2.

In feature representation and embedding module, we use an embedding layer to transform informative features into dense vectors. In order to get session sequence, we divide user behavior sequences into sessions in session division module. In session interest extractor module, we employ multi-head self-attention to reduce the influence of unrelated behaviors and capture the inner relationship between behaviors in the same session. In session interest interacting module, we use LSTM to get the feature interaction. At the same time, we use A-LSTM to model the representation of session interests and target ad. In prediction module, embedding of sparse features and session interests that we capture are concatenated and then imported into MLP. Finally, the softmax function is used to get probability that people click on the ad.

The loss for the auxiliary task can capture more interest representation and it can also enforce the states of the BLSTM module to effectively learn the user interests. Let *I*^*i*^ denotes the clicked interest sequence, and ${\hat{I}}^{i}$ denotes the negative sample sequence. *I*^*i*^[*t*] denotes the *t*-th vector for user *i* clicks. ${\hat{I}}^{i}[t]$ represents the vectors of without the *t*-th step. *T* denotes the number of user’s behaviors. The loss for the auxiliary task can be defined as:

$$L_{aux}=-\frac{1}{N}(\sum_{i=1}^{N}\sum_{t}^{T}\log\sigma (h_{t},I_{}^{i}[t+1])+\log(1-\sigma (h_{t},{\hat{I}}_{}^{i}[t+1])))$$ (13)

where σ is the sigmoid activation function and *h*_*t*_ is the *t*-th hidden state of the BLSTM network.The loss function is a negative log-likelihood function and is expressed as:

$$L=-\frac{1}{N}\sum_{(x,y)\in D}^{N}\left(y\log p(x\right)+(1-y)\log(1-p(x)))+\alpha *L_{aux}$$ (14)

where *D* denotes the training size *N*, *p*(*x*) denotes the probability that the user clicks on an ad. *α* denotes the hyper-parameter that is used to balance the interest representation and the prediction of the CTR.

## 4. Experiments

### 4.1 Experiments setting

#### Datasets

In this section, we conduct experiments on four datasets: Books and Electronics in Amazon dataset [42], two public datasets: Avazu and Criteo. The dataset is showed in Table 1. The datasets are randomly divided into three parts: training set (80%), validation set (10%) for adjusting hyper parameters and the rest 10% is for testing.

**Table 1:** Basic statistics of the datasets.

Dataset	Users	Items	Features	Samples
Books	67,282	52,933	50,775	306,287
Electronics	46,344	40,295	41,831	223,541
Avazu	86,971	76,349	130,748	804,312
Criteo	39,563	38,257	50,175	230,523

#### Evaluation metrics

We use three evaluation metrics in our experiments: AUC (Area Under ROC), Logloss and RMSE (Root Mean Square Error). The curve in AUC means the ROC [43], which is used to evaluate the performance of a two-class classifier. We believe that the larger the value of AUC, the better the performance of model. Logloss is applied to calculate the distance in a binary classification problem. The value of logloss is smaller, the performance of the model is the better. RMSE [44] can be defined as follows:

$$RMSE=\sqrt{\frac{1}{\left|T\right|}{\sum_{i}\left(y_{i}-{\hat{y}}_{i}\right)}^{2}}$$ (15)

where $y_{i}^{t}$ is the observed scores and ${\hat{y}}_{i}^{t}$ is the value of prediction, *T* is the testing set. Like logloss, we want to get smaller values.

#### Parameter settings

We set the size of the hidden state in the LSTM is 48. The different learning rates of 10^-4^,10^-3^,10^-2^,10^-1^ are used to test. Also, different number of neurons from 100 to 800 is employed.

### 4.2 Comparisons with different models

**Fig 3:**
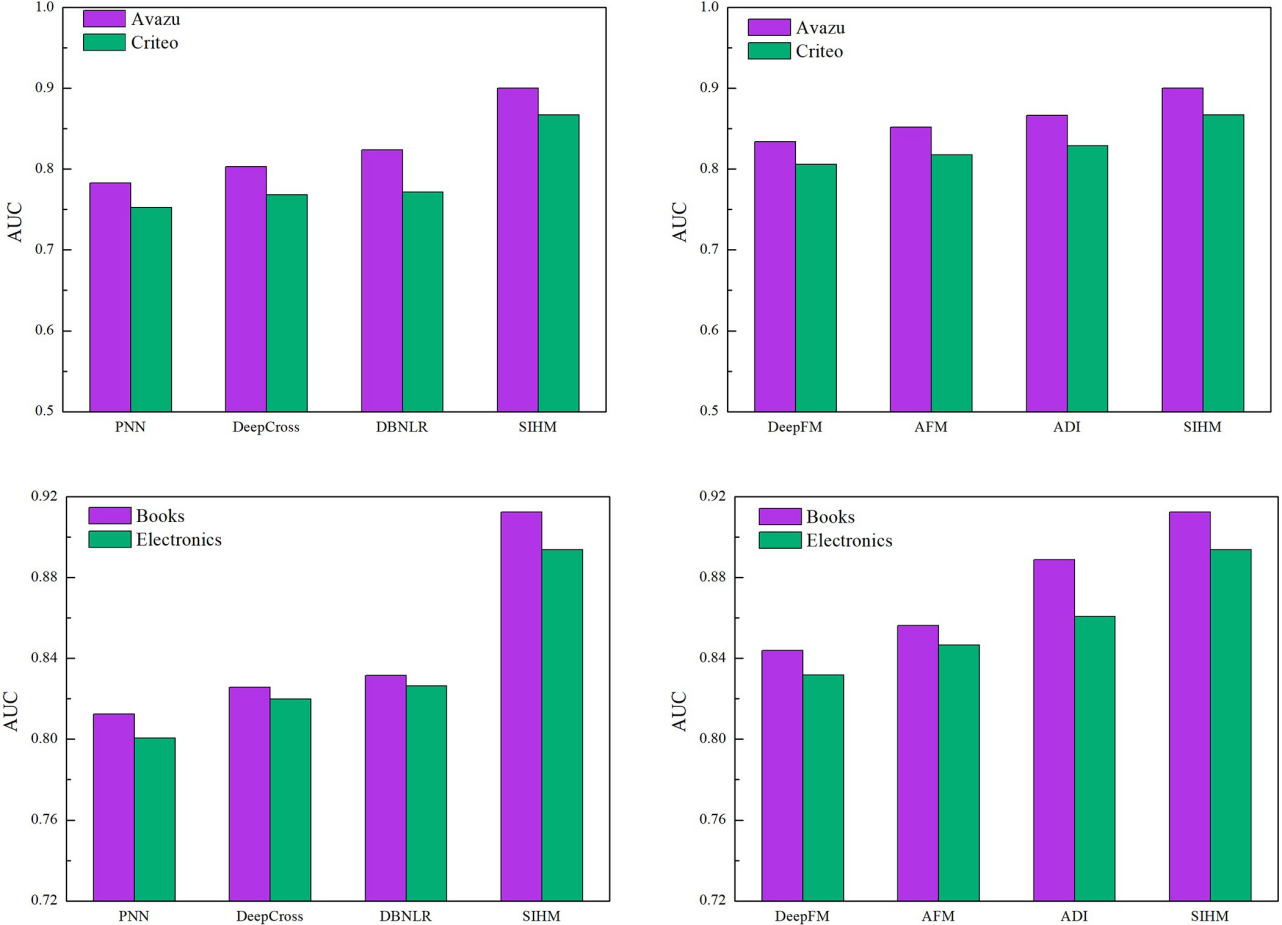
3-1 AUC performance comparison with other model. 3-2 AUC performance comparison with other model. 3-3 AUC performance comparison with other model. 3-4 AUC performance comparison with other model.

This section compares the SIHM model with some of the most advanced models currently in CTR prediction. In Fig 3, we can see the results of the different models for. Tables 2 and 3 show the value with logloss and RMSE respectively. The following aspects can be noted according to the comparison.

1. PNN introduces a product layer between embedding layer and full-connected layer, and uses neural networks to learn feature interactions automatically. However, the model ignores the low-order feature interactions, which are also important for CTR. So the PNN does not have better performance.
2. DeepCross is a model that automatically combines features to produce superior models. The important crossing features are discovered implicitly by the networks. DeepCross outperforms PNN, but the deep architecture is hard to optimize in training stage. DBNLR is a model for CTR prediction based on deep belief nets. The model combines the powerful data representation and feature extraction capability of DBN. At the same time, it uses LR to get the result of prediction. DeepFM is a new network framework that combines the FM and deep neural networks. The model can be trained without any feature engineering. So the model outperforms both DeepCross and DBNLR.
3. AFM is a CTR prediction model that can distinguish the importance of different feature interaction. As we all know, different feature interaction has different useful for results. The performance of AFM is better. This can be verified that using the attention mechanism can enhance performance of the model.
4. ADI captures interest evolving processes from user behaviors and gets higher prediction accuracy. However, the SIHM model performs better than others. The model uses multiple historical sessions to simulate the user’s sequential behavior in the CTR prediction task. We can see that SIHM model based on session interest can improve accuracy in all datasets.

**Table 2:** Overall CTR prediction for Logloss performance in different datasets.

Model	Logloss			
	Books	Electronics	Avazu	Criteo
PNN	0.1358	0.1436	0.2397	0.3732
DeepCross	0.1312	0.1403	0.2344	0.3654
DBNLR	0.1287	0.1374	0.2285	0.3527
DeepFM	0.1196	0.1325	0.2203	0.3490
AFM	0.1161	0.1277	0.2172	0.3375
ADI	0.1129	0.1202	0.2098	0.3224
SIHM	0.1043	0.1145	0.1935	0.3106

**Table 3:** Overall CTR prediction for RMSE performance in different datasets.

Model	RMSE			
	Books	Electronics	Avazu	Criteo
PNN	0.4716	0.4945	0.5219	0.5917
DeepCross	0.4635	0.4827	0.5163	0.5828
DBNLR	0.4528	0.4783	0.5078	0.5721
DeepFM	0.4503	0.4691	0.5023	0.5632
AFM	0.4481	0.4526	0.4985	0.5582
ADI	0.4205	0.4372	0.4871	0.5415
SIHM	0.4127	0.3937	0.4705	0.5267

### 4.3 Sensitivity analysis of the model parameters

We carry out an influence of different parameters in SIHM model, such as the epoch, the number of neurons per layer, and the dropout rate *β*.

**Fig 4:**
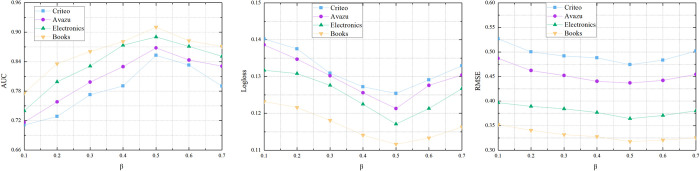
4-1 Performance comparisons w.r.t. the dropout rate *β*. 4-2 Performance comparisons w.r.t. the dropout rate *β*. 4-3 Performance comparisons w.r.t. the dropout rate *β*.

Dropout is the probability of neurons remaining in the network. We explore the value of *β* from 0.1 to 0.7. In Fig 4, we can see that the SIHM model performance better when *β* is properly set (from 0.4 to 0.7). However, with an increasing of the value of *β*, the performance of SIHM shows a downward trend. We choice the value of *β* is 0.5 in our experiment.

**Fig 5:**
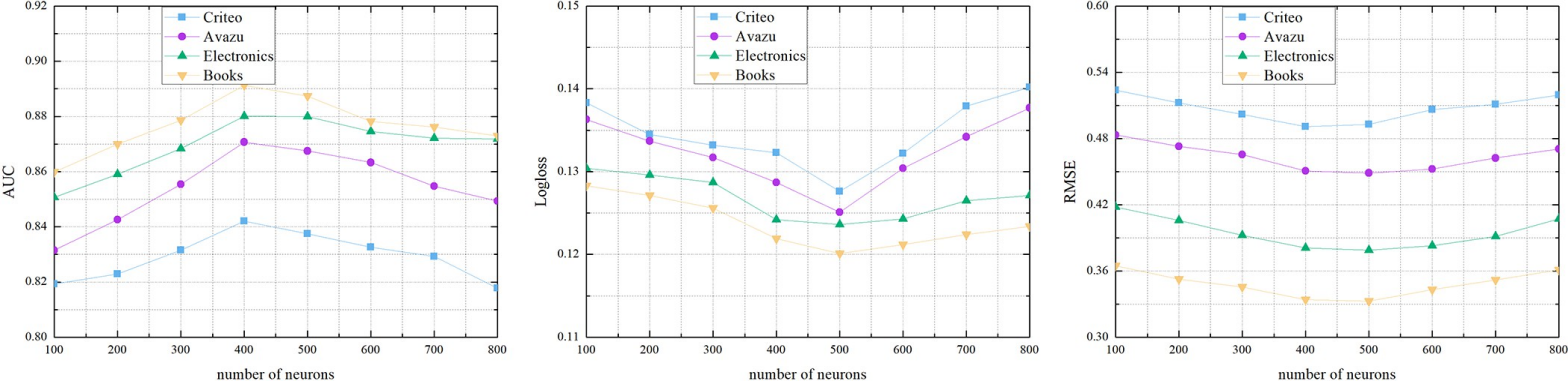
5-1 Performance comparisons w.r.t. the number of neurons. 5-2 Performance comparisons w.r.t. the number of neurons. 5-3 Performance comparisons w.r.t. the number of neurons.

When other factors remain the same, we study the effect of different number of neurons. We can see from Fig 5, the number of neurons has an impact on the accuracy of the model. As the number of neurons increases, the performance of the model decreases (from 600 to 800). The model develops an overfit problem. In this section, we set 400 as the number of neurons.

**Fig 6:**
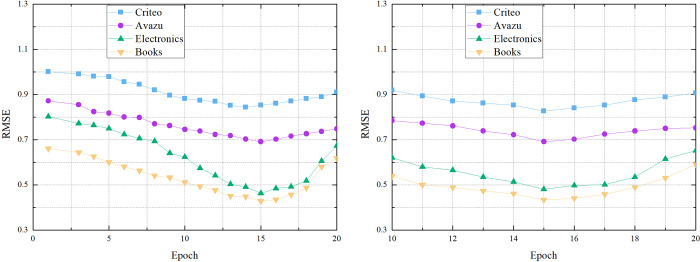
6-1 The effect of the epoch. 6-2 The effect of the epoch.

Fig 6 shows the effect of the epoch choice for the CTR prediction. Appropriate epoch makes the model get better prediction effect. It can be seen from Fig 6 that the model has better performance when the value of epoch is between 10 and 20. So we set the value of epoch to 15.

## 5. Conclusion

In this paper, we propose a hierarchical model based on session interest (SIHM) for CTR prediction. In order to get session interest, we divide the user sequential behavior into sessions and design session interest extractor module. To effectively capture session interest, we employ a self-attention network obtain an accurate expression of interest for each session. At the same time, the auxiliary tasks are employed for producing the interest state with the deep supervision strategy to learn the current hidden state. We use BLSTM to capture the interaction of different session interests. Then the attention mechanism based LSTM (A-LSTM) is used to aggregate their target ad to find the influences of different session interests. Finally, embedding of sparse features and session interests that we capture are concatenated and fed into MLP. The experiment demonstrates that the model achieves consistent improvements compared with the state-of-the-art models. In the feature, we will combine text features with image features [45] to build a CTR prediction model.
